# 

**Published:** 1886-01

**Authors:** 


DIED.—At Blooming Grove, Texas, January 4, Dr. Thomas Callaway, from the effects of an overdose of chloral; whether accidental or with suicidal intent is not known.

DIED.—We have room only to announce the death of Dr. W. A. East, of Halletsville, on December 9, of pneumonia. Dr. E. was one of the most distinguished physicians of Texas. More anon.

MARRIED.—At Independence, Texas, January 1, 1886, Dr. R. S. Johnson, Assistant Physician to State Lunatic Asylum at Austin, to Miss Maybell Dever, of Washington county.

MARRIED.—On the evening of December 30, ult. at the Cumberland Presbyterian Church, in Austin, by Rev. Mr. Poindexter, Dr. T. J. Bennet to Miss Amanda, eldest daughter of T. L. Hume, Esq., all of this city. A brilliant reception followed at the residence of the bride's parents.

I stood at the source of the majestic Trinity River;—the confluence of Clear Fork and West Fork. These streams drain, the one,—a prairie country, and, clear as crystal, it flows peacefully along its shady bed to its destiny; the other, a mountainous region;—and, more tumultuous and turbid, it rushes boldly amongst the rocks and crags with which its course is fretted. Meeting at this point, the blending is beautiful,—and the result, a majestic river of purity and strength; each tributary imparting something of its characteristics; -the one, giving its limpid purity and gentleness, receives increased vigor and velocity; and together they flow to their destiny, the Gulf,—each losing its identity in the whole.

So may these two young lives,—the one of virgin purity and gentleness, the other of manly beauty and strength, blend,—

and forming a union as beautiful, as grand, as peaceful, flow on to the great gulf of eternity, without a wave of sadness to stir, or a shade of sorrow to cloud their peaceful current. D.

N. B.—Our Dr. Merryman thinks "a Trinity" will likely result.

APOLOGETIC.—In consequence of overestimating our available space in the last form we are obliged again to omit several notices of books with which we have been favored, and also of Bartley's Test Case, which notices we had prepared. They shall appear in our next.

